# Items

**Published:** 1882-04

**Authors:** 


### Items.

THE following item in regard to Samuel Piercy, the actor, who died of small-pox a short time ago, is copied from the New York correspondence of the *Philadelphia Press;* "He was one of a half-dozen intelligent men I ever knew to be influenced by the crazy howls of the anti-vaccination fanatics. Jebb and Bergh and the rest of the mistaken lot had managed to convince him that the risks lurking in the preventive were worse than the dangers of the disease. Before leaving New York, a few weeks ago, he laughingly rejected the advice of friends who urged him to be vaccinated. He was a convert to the views of Jebb and Bergh, and he paid the penalty of martyrdom." —*Michigan Med. News.*

---

AMONG the graduates at the college commencements recently held in this city, were a number of doctors who had been in the practice for many years. Some had grown gray already in their calling. A number are leading physicians in the communities where they reside. After attending one course years since, they gradually grew into good practice and found it inconvenient to leave it for another course. While the recent law seemed to work harshly with some of these individual cases, yet the propriety of it was unanimously endorsed even by those thus put to some inconvenience. They yielded gracefully and only regretted that the measure had not been enacted years since.

---

By a private letter from a physician, residing in the southern part of this State, we are advised that some disastrous conseqences have followed the employment of the so-called "cone virus" which was recently used under the direction of the town authorities for the gratuitous vaccination of the citizens. Our correspondent states that the majority of the persons inoculated with this matter suffered from excessive inflammation of the arm, and "sloughing ulcers, deep and wide, with the most torturing pain, lasting from three to five weeks." In a number of the cases some of the axillary glands suppurated and sloughed out.

---

The committee of arrangements of the State Medical Association are preparing a programme for the entertainment of the members of that body who may attend the meeting to be held in this city on the 19th, 20th, and 21st of April, which will embrace every day of the session, so that the more serious work of the Association will be interspersed by seasons of social relaxation and enjoyment. The committee are assured of a large attendance and will spare no pains to render the occasion one of unusual pleasure and, it is hoped, of much profit.

---

Guy's Hospital, of London, and its management, have recently been severely criticised by English writers, lay and medical. A patient died from the effects of ten grains of morphine which was administered by the nurse through mistake, instead of ten grains of quinine as directed. The morphine was in one paper, probably not labeled, and was intended for the bladder, and not the stomach, of another patient. Some time before this a patient in the same institution lost his life through the carelessness of a nurse.

---

Bed bug in the Ear.—In the *Medical Brief*, Dr. J. T. Booth tells of a little child in whose ear an insect had taken quarters, thereby producing a commotion among the whole family, and a good deal of it with the child. A light was held near the ear, in order to obtain a view, when forth there stepped, in all his pride and glory, a stately bed bug, to ascertain the cause of the disturbance. He had been suspected of being a fly. Ordinarily, we believe, this insect runs from a light, rather than to it.

---

IN a recent paper upon the medico-legal aspects of injuries of the knee joint, Dr. Rueben A. Vance (*Ohio Med. Jour.*) made the statement "that he was cognizant of nearly fifty suits for malpractice now pending within a hundred miles' radius of Cincinnati, nearly all of them brought by impecunious parties against men of average ability who had accumulated sufficient means to tempt the cupidity of the plaintiffs and their accomplices."

Wonder if rival physicians were the accomplices of the plaintiffs?

---

"WE have received an Ephemeris of Materia Medica, Pharmacy, Therapeutics, and Collateral Information. By E. R. Squibb, M.D., E. H. Squibb, and Chas. F. Squibb, A. B., Brooklyn, N. Y." This, we are informed, is be distributed gratutiously, and whenever inclination and circumstances suggest. It may be issued "bi-monthly, quarterly, irregularly, or not at all." It asks for neither exchanges or subscribers. This surely is a happy-go-lucky platform.

---

THE New York Physicians Mutual Aid Association seems to be in a flourishing condition. We rejoice that it is so. The physician in good practice is always overworked; he rarely accumulates wealth, only just enough to raise his family so that they feel the need of a liberal support. If he dies, that family's future is not always an easy one. We feel that the principle of these mutual protective associations appeals to the physician with more force than perhaps to any other class.

---

THE Rev. Dr. Robt. Collyer, we learn from the *Medical Times*, thinks he is worth as a nurse just precisely 80,000 trained nurses; or, rather, modestly says his wife would so estimate him. Preachers ought to have too much sense to utter such trash just for the sake of a sensation, or for the sake of saying something that ignorant people may call smart. Next in value to a good doctor is a trained and faithful nurse.

---

The curing of ulcers, by grafting with pieces of sponge rather than with integument has been recently practiced to some extent. Some writers claim that the sponge becomes absorbed; others, that it becomes organized and provided with vessels and nerves. We want further testimony before subscribing to either view. While some men may well be termed sponges, we do not believe that either a whole man or a piece of one can be made of a sponge.

---

Dr. O. W. Holmes says he has known a practitioner—perhaps more than one—who was as much under the dominant influence of the last article he has read in his favorite medical journal as a milliner is under the sway of the last fashion-plate. The difference between green and seasoned knowledge is very great, and such practitioners never hold long enough to any of their knowledge to have it get seasoned.

---

Some physicians seems disposed now to foster an iodoform *craze.* This agent is being recommended for all conceivable forms of wounds and ulcers. Iodoform is sometimes a very useful local application in certain ulcers, but we trust it will not have such sway as has the carbolic mania.

---

What age but this one could have discovered men affected with puerperal fever? In *Revista Clinica di Bologna*, is related such a case. The wife was first suffering from the disease, the brute of a husband had intercourse with her and became inoculated, suffered a severe form of septicæmia, and died on the 17th day.

---

The physicians of the Old World must surely receive larger fees than we of this side of the water. Dr. Erasmus Wilson made it known that he would give fifty thousand dollars for endowing a chair of pathological anatomy in the University of Edinburgh. From this school he received his LL.D., and his father was a student there.

---

Dr. Roberts Bartholow, in the *Medical News and Abstract*, says that he prefers picrotoxine, one-sixtieth of a grain per day, as a remedy for the night sweats of phthisis. Before the introduction of this agent he recommended atropia in doses of one-thirtieth grain per day.

---

Polk County, in this State, is said to contain, near its southern border, a large quantity of Epsom salt. It is asserted that the salt "crops out all over the ground," and that a small potion of a solution containing earth obtained from that locality produces the characteristic effect of the salt.

---

Judging by some of the Society Proceedings the tupelo uterine tents, first brought before the profession by Dr. G. E. Sussdorf, formerly of this State, now of New York city, are used to considerable extent by some of the prominent gynæcologists.

---

Four deaths under chloroform have been reported recently as occurring in various parts of England. Ether may be slow; but it seems to us that chloroform, as the man said of the croton oil pills, is sometimes unnecessarily hasty.

---

It is claimed that the burr of a surgical engine, in an operation for removing diseased bone, as performed recently in Philadelphia, revolved only fifteen thousand times per minute—two hundred and fifty times per second.

---

Of fifty-six graduates of the Nashville Medical College, (Medical Department of the University of Tennessee,) but one was from Georgia. Our State is taking care of her own medical students better than ever before in her history.

---

THE *Medical Gazette* says that Sir Henry Thompson has become a convert to the views of Dr. F. N. Otis in regard to the calibre of the normal urethra, after having strongly opposed them when first announced. In the discussion that followed Dr. Otis' earlier investigation, we believe that both he and Sir Henry Thompson took extreme and opposite grounds. The calibre of the urethra is certainly much greater than the modest old ultra-conservative English view would allow; and, yet, a man doesn't like to bore a half-inch augur hole into every penis he may chance to handle. In the main, though, Dr. Otis' views are correct, but need a little toning down.

---

THE *Dublin Journal of Medical Science* quotes cases to prove that ingestion of cheese and gingercakes will produce severe illness. This is strange, very strange! It seems though, there were little cavities in the cakes (whose age made them worthy of veneration) and that the holes and not the cakes, did the work. These cavities were lined with fungi of the species *ascophora nigricans* (and others too numerous to invent names for); and the outside of the cakes were covered with cryptogamic vegetations, composed of *penicilium glaucum* and *ascophora mucor.* Now let the gingercake eater beware! We have fairly warned him of these ambushed enemies.

---

The *British Medical Journal* thinks that it is proved that the moderate use of opium in warm climates is a desirable habit. In the language of the old woman who tasted the flint-rock soup, "who would have thought it?"

---

THE *College and Clinical Record* charitably suggests as a reason why many medical practitioners do not read medical journals is because some of them cannot read at all!

---

IF any of our subscribers have Volume VIII, No. 1 of the *Atlanta Medical and Surgical Journal*, they will confer a favor on the Publisher by corresponding with him.

It is said that the effort to establish a Garfield Memorial Hospital in Washington is not meeting with a very brilliant degree of success.

---

Dr. Joseph Pancoast, emeritus Professor of Anatomy in Jefferson Medical College, died in Philadelphia, March 7th, aged 77.

---

Cannot some of the able practitioners of this state give the profession their experience in the treatment of fevers with cold water ?

---

Some man away up in Maine is writing of the Fasting Cure for Consumption. Truly, we say, "the sun do move."

---

Gray's Anatomy has been translated into the Chinese language, and published in six volumes, at Foochow.

---

It is recommended that muriated tincture of iron be given in capsules. This may prove a useful idea.

---

Dr. Roberts Bartholow is publishing some articles in the *Medical News* on variola, varioloid, and vaccinia.

---

Dr. Brown, of Detroit, highly recommends the use of hydrangea arborescens in renal calculi.

---

The Woman's Medical College of Baltimore is the last one.

